# MULTISCALE MODEL OF CRISPR-INDUCED COEVOLUTIONARY DYNAMICS: DIVERSIFICATION AT THE INTERFACE OF LAMARCK AND DARWIN

**DOI:** 10.1111/j.1558-5646.2012.01595.x

**Published:** 2012-07

**Authors:** Lauren M Childs, Nicole L Held, Mark J Young, Rachel J Whitaker, Joshua S Weitz

**Affiliations:** 1 School of Biology and School of Mathematics, Georgia Institute of Technology310 Ferst Dr, Atlanta, Georgia 30332; 2 Department of Microbiology, University of Illinois at Urbana-Champaign601 S. Goodwin Avenue, Urbana, Illinois 61801; 3 Thermal Biology Institute and Department of Plant Sciences and Plant Pathology, Montana State University307 Plant Bioscience Building, Bozeman, Montana 59717; 4 Department of Microbiology and Institute for Genomic Biology, University of Illinois at Urbana-Champaign601 S. Goodwin Avenue, Urbana, Illinois 61801; 6 School of Biology and School of Physics, Georgia Institute of Technology310 Ferst Dr, Atlanta, Georgia 30332

**Keywords:** Evolutionary biology, host–parasite interactions, immune defense, microbial ecology, viral evolution

## Abstract

The CRISPR (Clustered Regularly Interspaced Short Palindromic Repeats) system is a recently discovered type of adaptive immune defense in bacteria and archaea that functions via directed incorporation of viral and plasmid DNA into host genomes. Here, we introduce a multiscale model of dynamic coevolution between hosts and viruses in an ecological context that incorporates CRISPR immunity principles. We analyze the model to test whether and how CRISPR immunity induces host and viral diversification and the maintenance of many coexisting strains. We show that hosts and viruses coevolve to form highly diverse communities. We observe the punctuated replacement of existent strains, such that populations have very low similarity compared over the long term. However, in the short term, we observe evolutionary dynamics consistent with both incomplete selective sweeps of novel strains (as single strains and coalitions) and the recurrence of previously rare strains. Coalitions of multiple dominant host strains are predicted to arise because host strains can have nearly identical immune phenotypes mediated by CRISPR defense albeit with different genotypes. We close by discussing how our explicit eco-evolutionary model of CRISPR immunity can help guide efforts to understand the drivers of diversity seen in microbial communities where CRISPR systems are active.

The CRISPR (Clustered Regularly Interspaced Short Palindromic Repeats) system is a recently discovered type of adaptive immune system which defends against foreign genetic material, for example, plasmids and viruses (Mojica et al. 2005; Barrangou et al. 2007; Brouns et al. 2008; Deveau et al. 2008; Horvath and Barrangou 2010). Importantly, the CRISPR system is purported to be the means by which some bacteria and archaea evade viral infection and lysis in the environment (Andersson and Banfield 2008; Held et al. 2010; Heidelberg et al. 2009). As we describe below, the molecular details of how the CRISPR system operates and how viruses evade it are topics of intensive study. Nonetheless, the fact that hosts with an operative CRISPR system undergo directed changes to their genome with respect to the introduction of foreign genetic material poses a challenge to theoretical efforts to understand the basis for coevolutionary-induced diversification among hosts and viruses. Nearly all theories of evolutionary dynamics have in common two tenets of Darwinian evolution: first, changes to organismal genomes, for example, mutations, are random (Luria and Delbruck 1943; Lederberg and Lederberg 1953); second, success of organisms depends on their ecological fitness (Lande 1976; Geritz et al. 1997; Nowak and Sigmund 2004). The CRISPR system suggests that a new class of models are necessary to describe host–virus coevolution that lies at the interface of Darwinian and Lamarckian evolution (Koonin and Wolf 2009).

As noted above, the CRISPR system utilizes a form of genome-level imitation that permits a microbial cell to direct genomic changes that may be beneficial to its survival against invading elements (e.g., Sorek et al. 2008; Horvath and Barrangou 2010; Marraffini and Sontheimer 2010a; Vale and Little 2010). CRISPR loci have been identified in 40% of bacteria and 90% of archaea (Grissa et al. 2007). In brief, the CRISPR system works as follows: bacteria and archaea may have multiple CRISPR loci, containing a set of CRISPR-associated (Cas) genes and a repeat-spacer region (Sorek et al. 2008; Jansen et al. 2002; Makarova et al. 2009). This region has “spacers,” that is, genetic subsequences usually 20–50 nucleotides long that match to the protospacers found in extrachromosomal elements such as viruses, plasmids, and transposons, which are separated by repeats (Bolotin et al. 2005; Pourcel et al. 2005; Marraffini and Sontheimer 2010a). The repeat-spacer regions are transcribed as RNA. Mediated by the Cas proteins, these CRISPR RNAs confer immunity against viruses and plasmids by targeting homologous stretches of DNA and/or RNA. Successful recognition of foreign genetic material (i.e., via Watson–Crick base pairing with specific subsequences known as protospacers) can lead to repression and/or digestion of the foreign genetic material (Hale et al. 2009; Marraffini and Sontheimer 2010a). However, the precise molecular mechanisms for immunity or interference and acquisition of new spacers remains a focus of continued research (e.g., Haurwitz et al. 2010; Marraffini and Sontheimer 2010b; Hale et al. 2009; van der Oost et al. 2009). The host genome evolves by partial imitation of viral genomes (or plasmids) for which it has survived exposure (see Fig. 1B). In contrast, viruses that infect a host cell and avoid detection by the CRISPR system and other viral immunity systems evolve via undirected mutation (see Fig. 1A).

**Figure 1:**
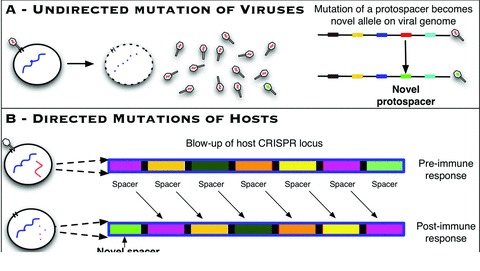
Schematic of the Darwinian and Lamarckian components of evolution in the CRISPR model. (A) Undirected mutation of viruses following successful infection leads to replacement with a novel protospacer within the viral genomes. New protospacers can occur anywhere in the protospacer set. (B) Directed mutation of hosts leads to inclusion of a novel spacer within the host genome. New spacers are added at the leading end. Note: We simplify the dynamics of spacer state change by assuming the maximum number of spacers per strain type is constant. When the maximum number is reached, the addition of a spacer at the leading end is accompanied by deletion of a spacer at the trailing end.

In this article, we introduce a model intended to capture the principles of host–virus interactions and coevolution via CRISPR immunity in an explicit ecological context. The model utilizes a multiscale approach to combine density-dependent ecological dynamics with evolutionary changes informed by the molecular rules of genomic change associated with the CRISPR system. We do so to further theoretical understanding of two questions. First, are the molecular mechanisms associated with the CRISPR system sufficient to lead to and maintain viral and host diversity and a complex host–viral community (Andersson and Banfield 2008; Heidelberg et al. 2009; Held et al. 2010)? Second, what are the evolutionary mechanisms by which directed and undirected mutational mechanisms remain in balance, in cases where coexistence is observed (Heidelberg et al. 2009; Held et al. 2010)?

A few other models have already made inroads in characterizing the effect that CRISPR defense may have on ecological and evolutionary dynamics. First, He and Deem (2010) utilized an immunological-based approach in which viral production is uncoupled from host density (and hence is less concerned with ecologically driven dynamics). That model concluded that spacers should be more diverse in the leading edge and also, that coexistence is possible among diverse strains. Second, Levin (2010) largely avoided the issue of coevolution, to examine ecological competition between strains that possess CRISPR immunity versus those that possess receptor-based immunity. The present model aims to unite these two perspectives: (1) by utilizing an explicit density-dependent ecological formalism for host–viral interactions, such as Levin (2010); (2) by examining the coupling between the ecological dynamics of strains and the evolutionary change of the genomic state of strains, such as He and Deem (2010). In so doing, the present model tracks the dynamics of both host and viral strain states as well as densities. A third model, by Haerter et al. (2011), also presents a similar approach, albeit with a focus on spatially-mediated interactions between viruses and hosts.

Here, we analyze coevolutionary-induced dynamics wherein hosts possess multiple spacers and viruses possess multiple protospacers. We observe that highly diverse assemblages emerge from low diversity initial conditions. The emergence and maintenance of diversity is due to a series of invasions by viruses and hosts. Diversity is maintained over the long term, but this diversity reflects the punctuated emergence of novel host and viral strains that have relatively short lifetimes. Hence, we find that populations are often highly similar on short time-scales but highly dissimilar over long time-scales. We also observe three types of evolutionary dynamics that drive short-term changes in our model: (1) invasion by rare strains with fitness advantages; (2) recurrence of rare, older strains that gain fitness advantages due to changes in the genetic states of other strains; (3) invasion by coalitions of strains with identical immune phenotypes but distinct genotypes. We note that coevolutionary driven diversification is not inevitable in such models, and point out conditions that favor CRISPR-induced elimination of viruses, which may be of interest in bioengineering applications. Additionally, we observe that CRISPR immunity is dominated by the most recently acquired spacers, an emergent feature of our simulations. Hence, we predict that only the first few spacers play a role in shaping the selective forces driving host–viral coevolution even when the spacer locus is comprised of many spacers. We show that the acquisition rate of new spacers is a stronger determinant of the complexity of the resulting community than is the failure rate of hosts to protect against viruses for which CRISPR immunity is already present. Finally, we discuss how this coevolutionary model framework can be utilized to help identify those factors driving CRISPR-induced coevolution in the environment.

## Models

The coevolutionary model presented here is comprised of three parts: (1) ecological; (2) molecular; and (3) evolutionary. The full model integrates these three components together to simulate the dynamic interactions between diverse hosts and viruses. In brief, host and viral densities are determined by ecological rules of interaction that include host reproduction and death, viral infection of hosts, and viral deactivation outside of hosts. The molecular component determines whether viral infection leads to host lysis, viral deactivation, or spacer integration. The evolutionary component introduces new host and viral strains and their genetic states (see Fig. 1). The dynamical steady state in the model is shaped by the assumptions built into the model as well as the quantitative values of model parameters (see below and Table 1). The details of these components and of the computational scheme used to implement them are described below. The model framework builds upon an earlier effort to study coevolutionary dynamics between bacteria and phages based upon the evolution of envelope receptor states within bacteria and tail fiber states within phages (Weitz et al. 2005). More broadly, multiscale eco-evolutionary models of this kind have been utilized elsewhere, for example, food web dynamics (Loeuille and Loreau 2005) and influenza-host disease dynamics (Koelle et al. 2006).

**Table 1:** Description and values of parameters in multiscale eco-evolutionary simulations. Details of how these parameters are integrated in the model are explained in the main text

Model component	Parameter	Meaning	Values
Molecular	*p*	CRISPR failure probability	10^−5^
	*q*	New spacer acquisition probability	10^−5^
Ecological	*r*	Growth rate (1/h)	1
	*K*	Carrying capacity (1/mL)	10^5.5^
	β	Burst size	50
		Adsorption rate (mL/h)	10^−7^
	*m*	Viral decay rate (1/h)	0.1
Evolutionary		Mutation rate	5 × 10^−7^
	ρ_*c*_	Density cutoff (1/mL)	0.1

### ECOLOGICAL COMPONENT

We consider a community comprised of hosts (either bacteria or archaea), viruses, and implicitly modeled resources. The densities of hosts and viruses change based on the following ecological events. First, hosts can divide given sufficient resources and they can also die. Here, we focus on a rather simplified ecological context, where resources are considered implicitly and host populations would increase to their carrying capacity in the absence of viruses. Viral populations increase (and host populations decrease) due to infection and lysis of hosts. Viral populations decrease due to spontaneous deactivation in the environment, a process thought to be characterized by a single time-scale (De Paepe and Taddei 2006). Virus populations also decrease due to unsuccessful infections. We denote *N_i_* as the density of hosts of strain *i* and *V_j_* as the density of viruses of strain *j*. Each host strain has a unique genomic state which we denote by *S_i_*, corresponding to the set of spacers it contains that confer it with CRISPR-derived immunity. Each viral strain has a unique genomic state which we denote by *G_j_*, corresponding to the set of protospacers it contains for which hosts may or may not be immune. Hosts reproduce at a maximum per-capita rate of *r_i_* with a carrying capacity of *K*. Viruses infect hosts at a rate ϕ_*ij*_. Here, we only consider two possible outcomes for a viral infection: (1) the host dies and new viral particles are produced; (2) the host disables the viral genome and (possibly) modifies its own genome in a directed fashion. Finally, viruses decay at a density-independent rate of *m*. Together these rules lead to the following dynamical equations:


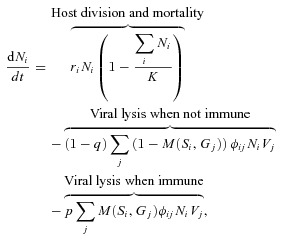
(1)


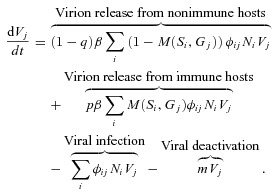
(2)

In these equations, *M*(*S_i_*, *G_j_*) denotes whether a host with spacer state *S_i_* is immune (*M*= 1) or not immune (*M*= 0) to a virus with protospacer state *G_j_*. The details of how such immunity is determined is explained in the following section, along with discussions of the meaning of the immunity parameters *p* and *q*.

### MOLECULAR COMPONENT OF CRISPR IMMUNITY

The immune defense of a host to viral infection is based on sequence matches between host and viral genomes. The immune state of a host is denoted as *S*= (*s*_1_, *s*_2_, …, *s_u_*) where *s_i_* is the *i*th spacer of *u* spacers in the CRISPR locus. In reality, multiple CRISPR loci may exist within a given host, however, here we only analyze a single locus. We simplify the dynamics of spacer state change by assuming the maximum number of spacers per strain is constant and that spacers are always added to the leading end. When the maximum is reached, the addition of a spacer to the leading end is accompanied by deletion of a spacer at the trailing end. Spacers are drawn from protospacers, that is, small subsequences within the viral genome. As such, we denote the genomic state of the virus relevant to CRISPR immunity as *G*= (*g*_1_, *g*_2_, …, *g*_*v*_), where *g_j_* is the *j*th protospacer of *v* protospacers in the virus. Throughout this analysis, we consider all undirected mutations to be drawn from an infinite number of alleles. This assumption is supported by studies that suggest that only a single base pair mismatch undermines CRISPR immunity (Barrangou et al. 2007). Other immunity models may follow from relaxing this condition. Here, CRISPR immunity is defined as follows:


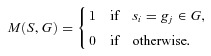
(3)

In words, *M*(*S*, *G*) = 1 if the spacer set in the host includes at least one perfect match to a protospacer in the virus, otherwise *M*(*S*, *G*) = 0. The CRISPR immune mechanism is not perfect (Barrangou et al. 2007). We expect errors will be of two types, false negatives and false positives. For false negatives, the CRISPR system may not identify a viral genome even though it possesses a spacer which matches a protospacer in the virus. For false positives, the host may randomly acquire a spacer matching a viral protospacer during an interaction (see below) which brings immunity along with it. Both CRISPR and non-CRISPR mechanisms may be involved. We model these types of errors quantitatively as follows. If *M*(*S*, *G*) = 1 (the host is immune to the virus via CRISPR defense), then two events can happen: (1) immune defense with probability 1 −*p*, via which the host survives and the virus is eliminated; (2) stochastic failure with probability *p*, via which the host is lysed by the virus leading to a burst of progeny viruses. If *M*(*S*, *G*) = 0 (the host is not immune to the virus via CRISPR defense), then two events can happen: (1) host lysis with probability 1 −*q* and subsequent burst of progeny viruses; (2) host survival with probability *q* in which the virus is eliminated. We assume that both *p* and *q* are small, that is, *p*, *q*≪ 1, as described in Model parameters below.

### EVOLUTIONARY COMPONENT

Undirected mutations of viral protospacers can occur upon successful infection of a host. Viruses that evade CRISPR defenses and other host defenses exploit host cellular machinery and produce β virions (see eq. (2)). Errors in replication can lead to the modification of one of the protospacer alleles in a given virion with a per-allele probability μ (e.g., the allele indicated by an arrow in Fig. 1A). The undirected mutation of a virus protospacer set is denoted as 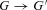. Note that in implementing the model, we do not consider simultaneous mutations or recombination mechanisms as means for introducing variation in protospacer states. Hence, if viruses are produced at a rate *b* per unit time in the entire system, then the expected number of mutations per unit time is *b*μ*v*, where *v* is the number of protospacers per virus. The stochastic timing of these events is determined via random sampling from an exponential distribution with mean time between mutations of 1/(*b*μ*v*). Due to changes in host and viral populations that affect the viral birth rate, this rate is recalculated during the simulation (see Supporting Information for details).

Directed mutation can occur when a host identifies and integrates a new protospacer into its CRISPR locus, where it is denoted as a spacer (see Fig. 1B). During every host–viral interaction, there is a small probability *q* of acquiring a new spacer through uptake of a protospacer. Depending upon the previous immune state of the host, the addition of a protospacer may change the immune state of the host with respect to the identified and imitated genotype. In other words, the host will become immune to all viruses which contain the protospacer that was integrated into *S*′ as a spacer. If the host already contained a matching protospacer, this new spacer does not provide any additional immunity. If a host did not previously contain a matching spacer, the newly added protospacer allows the host to survive. This often leads to a selective advantage for this host strain. To summarize: protospacer integration to the host can occur with rate *q* during any unsuccessful attempt by a virus to infect a host, regardless if the host previously had immunity to the attacking virus. Recall that the addition of a spacer at the leading edge is accompanied by the loss of a single spacer at the trailing edge of the CRISPR locus when the locus has a maximum number of spacers considered (eight in our simulations). Additionally, note that a host need only differ by a single spacer from all other host strains to be considered its own strain. All novel mutant strains are introduced in the simulation, regardless of whether they have a selective advantage or not.

### SIMULATION PROTOCOL

We begin our simulations with a single host strain and a single viral strain along with their respective spacer and protospacer states. Our initial host strain is susceptible to the initial viral strain and, thus, does not contain a spacer matching a protospacer of the virus. We simulate the ecological and molecular interactions of the hosts and viruses (see eqs. 1–2) deterministically using ode45 in Matlab. Population densities change until: (1) a host or virus strain goes extinct; (2) a mutation event occurs, either of the undirected (viral) or directed (host) type; or (3) the simulation reaches a defined time point for data output and recalculation of mutation rates that occur at periodic intervals. When any of these events occur, the simulation is paused and the strain mutation rates, which depend on the continually varying strain abundances, are recalculated (see Supporting Information for details.). Note that we use the term “mutation” here to denote the insertion of a sequence into the CRISPR locus of a host or the change in sequence of a viral protospacer. This process is repeated until one of the following occurs: all host strains go extinct, all viral strains go extinct, or the simulation reaches the maximum running time (generally 2500 h in the model). Simulations are run with 100 replicates, unless the computation per replicate is excessive in which case 75 or 25 replicates are used.

Strain extinction occurs when the population density of a strain falls below our critical population threshold, ρ_*c*_; this acts as an absorbing state for strains. Through an event function in ode45, the simulation is paused, and the system of ordinary differential equations (ODEs) is reduced by removing the equation for the strain which has fallen below the cutoff. Mutation events can occur upon replication by viruses (via an undirected mechanism) and upon virus infection of a host (via a directed mechanism) (see Fig. 1). Mutational events cause an addition of a new strain and thus the addition of a new ODE to the system. All mutant strains are given an initial density 10% greater than ρ_*c*_. The time until the next mutational event is calculated using the Gillespie algorithm. Since the time to the next mutational event is stochastic, replicate simulations will not give identical results. In the case of viral mutation, a given strain of virus is randomly selected to undergo a mutation event with probability in proportion to the instantaneous growth rate of that strain. Similarly, in the case of host acquisition of a spacer, a given host strain is randomly selected to acquire a new spacer in proportion to its instantaneous rate of successful defense events. Data output occurs at regular intervals throughout the simulation. After each event—strain extinction, strain mutation, or data output—the strain mutation rates are recalculated. Additional details of the simulation procedure are found in the Supporting Information.

### MODEL PARAMETERS

The choice of model parameters will vary depending on the CRISPR system of interest. In general, we consider ecological parameters typical of *Escherichia coli* and its phages (De Paepe and Taddei 2006) and molecular parameters consistent with small error rates in CRISPR immunity, *p*≪ 1 and *q*≪ 1. The value of *p* is based on work in *Streptococcus thermophilus* for which *p* can be considered an efficiency of plating of viruses on CRISPR immune hosts and can range from 10^−4^ to 10^−7^ (Barrangou et al. 2007). The value for *q* is based on work in the same system for which acquisition of resistance to virulent bacteriophages occurs rarely, *q*≍ 10^−6^ (Barrangou et al. 2007; Horvath et al. 2008; Deveau et al. 2008). Note that in this model, the value *q* denotes the successful integration of a novel spacer, and hence, directed mutation of the host.

Given the variation inherent in viral and host dynamics, we further restrict our attention to model parameterizations which obey the following two conditions: (1) viruses eventually die out when infecting immune hosts; (2) viruses coexist with nonimmune hosts. Given small error rates and large burst sizes and the definitions of the ecological and molecular components, these conditions can be written compactly as: 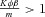 and 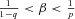 (see the Supporting Information). Note that actual ecological parameters remain poorly known for all but the most well studied of laboratory host–virus systems. Furthermore, when selecting hosts and viruses to model, we note that parameter choices are not independent. For example, viral mortality rates and their production rates (burst size divided by latent period) are positively correlated for some phages (i.e., phages which produce more virions degrade faster and those which produce fewer virions are more stable) (De Paepe and Taddei 2006). Parameter values that are used as baselines for all simulations (unless otherwise noted) are listed in Table 1.

Though the model framework can handle arbitrary numbers of spacers and protospacers, we focus computational efforts on cases when *u*≤ 8 spacers and *v*≤ 10 protospacers. The choice of spacer and protospacer number ensures that the number of spacers is less than the number of protospacers, as is the case biologically. Further, our choice is made as a concession to efficient numerical simulation of the model, whose simulation time increases with protospacer number. Computational time and power also limits the number of replicates it is feasible to consider.

## Results

### VIRAL AND HOST DIVERSIFICATION IN A MULTIPLE SPACER, MULTIPLE PROTOSPACER MODEL

Here, we examine the dynamics of a host–virus community in which each host possesses multiple spacers and each virus possesses multiple protospacers. We find that hosts rapidly acquire CRISPR immunity through directed incorporation of spacers. Viruses mutate randomly at one of multiple protospacer sites so that not all viral mutations are immediately beneficial. Nonbeneficial viral mutations may arise if viral mutations occur at protospacer sites for which no host possesses CRISPR immunity. In contrast, viral strains that have mutations of specific protospacer sites for which hosts have CRISPR immunity may gain some fitness advantage. In this model, host types emerge that are CRISPR immune to multiple (but not necessarily all) viruses and, likewise, viral types emerge that infect some (but not necessarily all) hosts (see Fig. S1).

**Figure 2:**
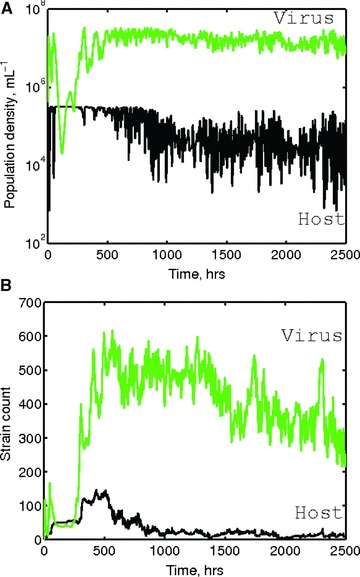
Dynamics and diversification of multiple spacer–protospacer model (eight spacers, 10 protospacers). (A) Viral population dynamics (green online) and host population dynamics (black) show that population densities undergo fluctuations. (B) Viral strain count (green online) and host strain count (black) show the diversification into multiple host and viral strains. These graphs show results from a single representative simulation (out of 100 replicates).

In this system, complex coevolutionary dynamics unfold (see Fig. 2A). Note that since the host generation time is ∼1 h in this model (see Table 1), we will refer to dynamics in the equivalent scale of generations. In the first 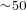 generations, the densities of hosts and viruses oscillate around a dramatically increasing average. The number of host and virus strains initially increases, often exceeding dozens and sometimes hundreds of strains (see Fig. 2B). After the system passes transient dynamics, the virus population exceeds the host population in both density and strain count (see Fig. 2). Typically by 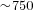 generations, the CRISPR spacer locus of all host strains has acquired a full array of eight spacers. Subsequent strains also have a full locus. Thereafter, the average number of host and virus strains (from 100 replicates) remains relatively constant, although the density and abundance of any particular strain in any particular simulation changes dramatically. Cross-correlation analysis will be considered in a follow-up work given the interest in phase lags of consumers and resources within eco-evolutionary dynamical systems such as this (Yoshida et al. 2007).

**Figure 3:**
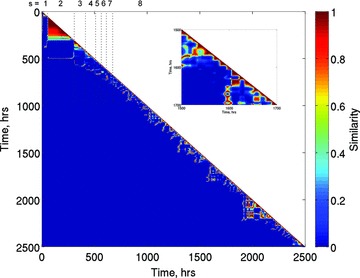
Incorporation of spacers causes changes in host population size and host population content. Ecological similarity between the whole host population at two time points using the Morisita–Horn index which takes into account both abundance and type (see eqs. 4–5). Time intervals of 2 h are used. The color bar indicates similarity from blue (low similarity) to red (high similarity). The diagonal is the comparison of one community against itself and hence has perfect similarity (dark red). Communities significantly separated in time are blue indicating no similarity (see bottom left of the figure). The vertical bars indicate an increase in the average number of spacers per host. The average number of spacers, *s* is marked above the graph and saturates at a maximum of *s*= 8. The inset is an enlarged version of *t*= 1500 to *t*= 1700. This graph shows results from a single representative simulation (out of 100 replicates).

A viral strain does not necessarily suffer an extinction when a host acquires immunity to that viral strain, because there are other host strains present which that viral strain may be able to infect (see Fig. S1). However, evolutionarily induced extinction of viruses does occur when undirected mutation does not generate viral strains that can evade CRISPR immunity to individual hosts or a coalition of hosts. For example, if μ, the mutation rate of our viruses, is too small (e.g., μ < 10^−8^ given parameters utilized here) then there is the possibility that there are too few viral infections that lead to novel viral strains with the ability to evade CRISPR immunity (see Table S1). Such viral extinctions can also be ecologically induced even for larger values of μ. This finding is important as it points out that multiscale eco-evolutionary CRISPR models may be appropriate for the study of host–virus dynamics in natural environments (where coexistence may be of interest) or host–virus dynamics in industrial contexts (where viral elimination may be a goal).

### DIRECTED MUTATION OF HOSTS CHANGES HOST POPULATION SIZE AND CONTENT OVER THE LONG TERM

We find that the strain composition changes over the course of a simulation, despite the maintenance of high diversity throughout. In other words, strains arise, exist for some period of time typically between 0 and 400 h, and are lost (see Fig. S2). To quantitatively compare the strain composition of the host population over time, we employ the Morisita–Horn similarity index (Wolda 1981) which defines the similarity of communities at times *t*_1_ and *t*_2_ taking into account the types of strains and their abundances:


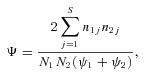
(4)

where


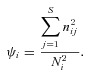
(5)

In equations (4–5), *S* is the total number of unique strains found in one or both communities, *N_i_* is the total number of individuals in community *i*, and *n_ij_* is the number of individuals of strain *j* in community *i* (Wolda 1981). Further, note that ψ_*i*_ is the similarity of community *i*, and is equivalent to the probability that two randomly chosen individuals in that community are of the same species. The index functions as follows: the index is zero when there is no overlap of strain types between time points; it is low (near zero) when there is some overlap of strain types but at vastly different abundances; it is high (near one) when there is overlap of many strains and these strains exist at similar abundances; it is one when there is exactly the same strains at exactly the same abundance (i.e., comparison of a time point to itself).

In the absence of mutation, our choice of parameters allows hosts and viruses to stably coexist (see the Supporting Information). Although such a case is biologically unfeasible, it is instructive to consider theoretically. In such a case without mutation, the content, or strain types, of the population would not change, but the population density of viruses and hosts would change over time, such that the Morisita–Horn index, Ψ, would never reach zero so long as hosts and viruses persist.

For our multiscale model that includes both directed and undirected mutation, we calculate Ψ for all pairs of recorded time points in our simulation, leading to the matrix in Figure 3, where red denotes high Ψ and blue denotes low Ψ. As shown in Figure 3, we find rapid turnover of host strains over time-scales that span the simulation. Directed mutation of a host can provide the new host strain with immunity to a greater proportion of the virus population, allowing the host strain to increase in relative abundance. We observe repeated instances in which the host population is similar to itself over a short period of time (see insert to Fig. 3). Instances of short-term similarity are typically correlated with oscillations in host density defined by our ecological model parameters (the small red triangles near the diagonal in Fig. 3). We observe that both hosts and viruses have a short lifetime (see Fig. S2).

Similarity between populations can extend beyond short oscillation time scales of invasion when there is near-dominance by a limited number of host strains as shown in the Figure 3 insert. We do not observe a fixed time scale or interval for host populations to be maintained with nonzero similarity. Eventually, the derived strains out compete their ancestors, causing these older strains to fall below the threshold and be removed from the simulation. Throughout the simulation, the fact that populations are not similar to populations at much later times implies that the subsequent populations are dominated by novel evolved hosts rather than recurrences of prior hosts over the simulation time scale of 2500 generations (see the large blue section in the bottom left of Fig. 3).

### INCOMPLETE SWEEPS AND BOUTS OF DIVERSIFYING EVOLUTION DRIVE CHANGES IN POPULATION COMPOSITION IN THE SHORT TERM

Here, we focus on evolutionary dynamics occurring on the short term. We note that at any point in the simulation, there are only a few dominant host and viral strains whereas many diversified strains exist at low abundance (see Figs. S1 and S3). Throughout the simulation, novel host variants evolve from two types of ancestor strains: highly abundant strains, because they make up more of the population and thus have a greater chance of interacting with viruses and gaining a spacer via directed mutation; and less-abundant strains that already have immunity to an infecting virus because they have the possibility to acquire an additional spacer during successful CRISPR immune defense to viral infection. The second mechanism for generating new strains occurs infrequently because few viral–host interactions occur among rare strains.

Because viral mutation is undirected, the majority of new viral mutants do not have significantly increased fitness because they have not mutated the specific protospacer that dominant host strains have immunity to. On average, only 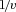 mutations, where *v* is the number of protospacers, will produce a viral mutant that alters the dominant host immunity. In contrast, because of the CRISPR mechanism of directed mutation, hosts respond directly to viral selection.

We find that the dominant host strains die out and are replaced by new strains (as defined in our model) with different characteristic evolutionary dynamics: (1) incomplete selective sweeps; (2) negative frequency-dependent selection; and (3) clonal competition of strains with the same immune phenotype. Based on these dynamics, one of these three types of strain cohorts dominate at any one time: (1) a newly evolved resistant strain, (2) an older strain maintained in the population at low abundance, or (3) multiple strains resistant to similar viral subsets, albeit with different spacers.

With the exception of early time points in our simulation, we do not observe complete sweeps with a single strain eliminating all others; the time-scale of evolution and ecology is mixed such that multiple strains nearly always coexist in our simulations. Further, we find that in the different evolutionary dynamics described above, hosts rarely incorporate the newest protospacer (see Fig. S4). Rather, the host spacer state reflects a history of the ecological success of viruses and their protospacers, but not necessarily a chronological history of protospacer appearance. Below, we describe each of these evolutionary dynamics in greater detail.

First, incomplete selective sweeps are expected given the fact that ecological and evolutionary time scales are mixed in this model. An incomplete selective sweep occurs when a host strain evolves that is resistant to viral types, expanding its range of resistance. Such a host strain usually evolves from a host strain that was abundant, as noted above. If this strain has a significant advantage (i.e., maintains a spacer that matches the dominant viral variants), it can grow to dominate in abundance during the next period of high density (see red, green, and dark blue curves in Fig. S3). In response, a viral variant that can infect the newly derived host has a competitive advantage and quickly increases in frequency. This is consistent with the arms race dynamic of coevolution through successive rises and falls of newly evolved host and viral strains (Buckling and Rainey 2002).

**Figure 4:**
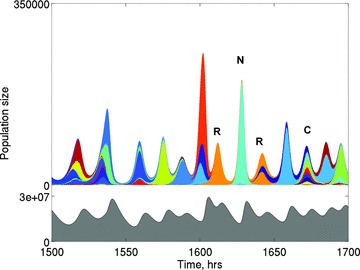
Proportion of host strains in the population. Host strains (independent colors—colors repeat when not directly touching) are born into the population and increase in size over time. The total height of the colored area is proportional to the population size and the vertical height of each color within the colored area is proportional to the percent of the population comprised by each strain. Strains first appear in the middle of the color that is their parent strain. Some novel strains (i.e., light blue at *t*≍ 1625 denoted by **N**) rapidly become the dominant strain. At times, multiple hosts emerge as coalitions and comprise significant portions of the population (i.e., at *t*≍ 1675 denoted by **C**). Finally, recurrence of strains can be observed (orange peaks at *t*≍ 1610 and *t*≍ 1640 correspond to the same strain denoted by **R**). Only host strains comprising at least 1% of the population are included. The total viral population density is shown in the lower panel. This graph shows results from a single representative simulation (out of 100 replicates).

Rare, older host strains may also rise to dominance at periods of high host density in a manner consistent with negative frequency-dependent selection, albeit for mechanisms that are predominantly evolutionary in nature (see Fig. S3 light blue and magenta curves and the two orange peaks in Fig. 4, note that colors should be interpreted separately for these two figures). In contrast to the selective sweep model, these strains are not derived directly from the previously highly abundant strain. Instead their fitness advantage arises because viral populations to which they are immune grow in number when targeting a different high-abundant nonimmune host. Likewise, viral populations to which they are not immune may decrease in number when targeted by CRISPR immunity of abundant hosts. Together, these mechanisms may have a secondary effect of decreasing viral-induced mortality of this rare, older strain (which possess a different array of spacers). Decrease of viral-induced mortality leads to the population expansion of the rare, older strain. As pointed out above, we do not observe any host strains that persist over the time course of the entire simulation.

Finally, we also observe periods of high host population density in which there is not a single dominant host strain but a coalition of host strains that rise together to high abundance. This can result from multiple host strains each gaining a new spacer that matches a different protospacer in a dominant virus or viruses (i.e., around 1675 h in Fig. 4). These strains are phenotypically nearly identical but differ genotypically. These coalitions fall in abundance due to the rise of a divergent virus that does not possess any protospacers that match any of the newly added spacers. The rise of a coalition of hosts can also result from a set of rare strains already existing in the population in a manner similar to what is described above.

### IMMUNITY OF HOSTS IS CONTROLLED BY RECENTLY ACQUIRED SPACERS

**Figure 5:**
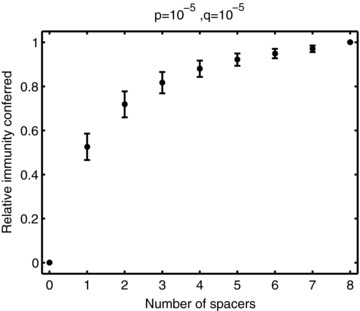
Most recently acquired spacers provide greatest immunity. Relative immunity conferred by the newest *n* spacers in the locus compared to the immunity from the full locus of eight spacers. Mean (circles) and standard deviation (error bars) were computed for 100 replicates averaged over the time points after the locus is filled with spacers. Immunity is determined by calculating what percentage of the viruses the most recent *n* spacers from all hosts can match, where *n*= 1, 2, …, 8. Relative immunity is the percentage of viruses that the most recent *n* spacers from all hosts can match compared to the percentage of viruses the full spacer locus (in our case eight spacers) matches. The majority of the immunity is provided by the first spacer and more than 80% immunity is provided by the first three spacers.

A recent coevolutionary CRISPR model of hosts and viruses found that spacers are more diverse in the leading position of a CRISPR locus (He and Deem 2010) in agreement with observation (Horvath et al. 2008; Held et al. 2010). This diversity is consistent with the mechanism by which spacers are inserted at the leading position of the locus. Our model demonstrates that not only are the leading spacers more diverse, but they also emerge as the most important spacers for providing the host strains with CRISPR immunity (see Fig. 5). For each time point, immunity is determined by calculating the percentage of the total viral population to which hosts harbor matching spacers. For example, the immunity provided by the first two spacers measures what percentage of all viruses the first two spacers of all host strains match at a particular time point. Relative immunity is the immunity calculated for particular sets of spacers compared to the immunity calculated for the full spacer locus (eight spacers in our case). Average values are computed over all hosts across all time points after the locus is full of spacers. This measurement indicates which of the spacer positions are most important for providing CRISPR immunity. We find that the first (and most recent) spacer of the locus contributes the greatest to the immunity of that locus, relative to the immunity provided from the entire locus. The contribution of subsequent spacers decreases such that only the first five loci are required to provide >90% of immunity, on average. Hence, the oldest spacers contribute insignificantly to the immunity of the locus (see Fig. 5). Although the increased diversity of leading spacers was previously known, both theoretically and empirically, until this model, it was not clear how important the recently acquired spacers were to CRISPR immunity. The emergent property from our model strongly supports the hypothesis that recently acquired spacers contribute substantially to CRISPR immunity, and moreover, predicts that they are sufficient for the CRISPR immune response, regardless of spacer identity at the tail end of the locus.

### DEPENDENCE OF COEVOLUTIONARY DIVERSIFICATION ON THE CRISPR IMMUNITY PARAMETERS

The molecular component determines whether viral infection leads to host lysis or viral deactivation. The errors associated with CRISPR immunity appear in the model through the constants *p* and *q*. Recall that *p* represents the stochastic failure of a host with CRISPR immunity to recognize an invading virus. Further, recall that *q* represents the acquisition rate of spacers by hosts.

**Figure 6:**
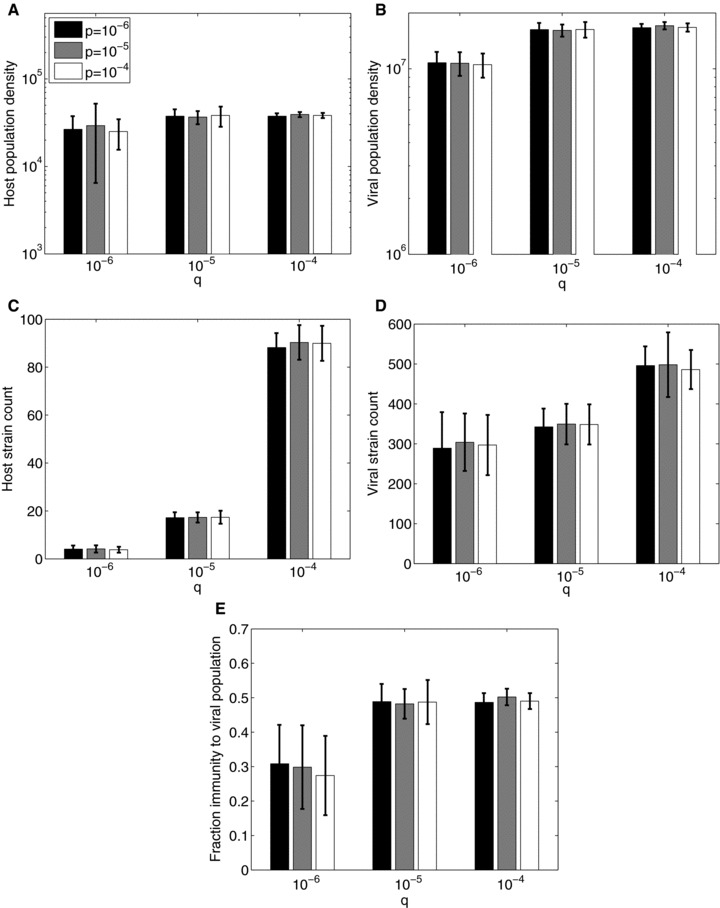
Population dynamics are more influenced by changes in the host spacer acquisition rate (*q*) than stochastic failure of CRISPR immunity (*p*). Stochastic failure of the CRISPR system when the host is immune, *p*, and host spacer acquisition rate, *q*, are varied from 10^−6^ to 10^−4^. Values of *q* are grouped on the x-axis. Values of *p* have identically colored bars (black represents *p*= 10^−6^; gray represents *p*= 10^−5^; white represents *p*= 10^−4^.) For all values of *p*, bars for *q*= 10^−4^ represent the median of 25 replicates, bars for *q*= 10^−5^ represent the median of 75 replicates, and bars for *q*= 10^−6^ represent the median of 100 replicates. Lines represent standard error. As *q* increases, host population density (A) is unchanged, viral population density (B) increases, host strain counts (C) increase, viral strain counts (D) increase, and the fraction of the viral population the hosts are immune to (E) increases.

**Figure 7:**
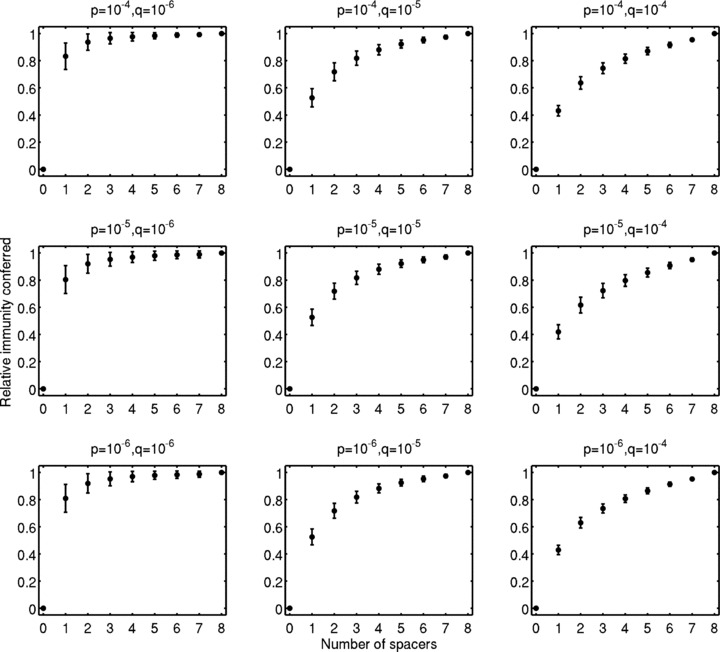
CRISPR Immunity is more influenced by changes in the host spacer acquisition rate (*q*) than stochastic failure of CRISPR immunity (*p*). Relative immunity conferred by the newest *n* spacers in the locus is compared to the immunity from the full locus of 8 spacers. Mean (circles) and standard deviation (error bars) were computed for all replicates averaged over the time points after the locus is filled with spacers. Immunity is determined by calculating what percentage of the viruses the most recent *n* spacers from all hosts can match, where *n*= 1, 2, …, 8. Relative immunity is the percentage of viruses the most recent *n* spacers from all hosts can match compared to the percentage of viruses the full spacer locus (in our case 8 spacers) matches. Values of *p* and *q* vary from 10^−6^ to 10^−4^. For all values of *p*, graphs for *q*= 10^−4^ include 25 replicates, graphs for *q*= 10^−5^ include 75 replicates, and graphs for *q*= 10^−6^ include 100 replicates.

Here, we consider the effect of varying values of *p* and *q* (around experimentally observed values) on the outcomes of the multiscale coevolutionary model. In varying these parameters, we considered values of *p* and *q* ranging from 10^−6^ to 10^−4^. We ran simulations using all nine possible combinations of *p* and *q* values in this range separated by a factor of 10, that is, *p*= 10^−6^, 10^−5^, 10^−4^ and *q*= 10^−6^, 10^−5^, 10^−4^. We find that altering *p* within this range has almost no effect on the dynamics in the simulations. The reason why *p* is not a major driver of dynamics at small values can be understood by examining equations (1–2). Small values of *p* increase lysis rates, albeit multiple orders of magnitude less than lysis rates of hosts that are not CRISPR immune. Hence, viral lysis is driven by the interaction of hosts and viruses for which CRISPR immunity is not present, whereas changing *p* only affects viral lysis in those interactions of hosts and viruses for which CRISPR immunity is present. However, varying *q* significantly modifies the complexity of communities even at small values. Higher values of *q*, corresponding to more rapid acquisition of spacers, lead to a high number of host strains without significant change in the host population size (see Fig. 6A, C). We expect more host strains with higher *q* because host strains are distinguished by their spacer states, which evolve more rapidly. In fact, only one spacer needs to be different to be considered a different strain. At higher values of *q*, viral strains, on the other hand, have a high number of strains and high population size (see Fig. 6B, D). This trend of increasing viral population size as the hosts can more easily acquire spacers may at first seem counterintuitive because it implies that viral population size is not a monotonically decreasing function of *q*. When *q*= 0, there exists a single susceptible host, and the viral population has the steady-state value 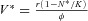 where 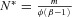 (see Supporting Information). When *q*= 1, all viral strains will be eliminated. However, when *q* is increased slightly above zero, we find that viral density increases. Viral density increases result from a greater ability of viruses to replicate, either because there exist more host strains that lack immunity or because the host strains that lack immunity have larger populations. Note that multistrain Lotka–Volterra models with fixed carrying capacity predict that viral density increases with the number of host strains (see Supporting Information). In the case of increasing *q*, we expect increases in the number of host strains and thus the possible secondary effect of increasing viral diversity and density (see Supporting Information). Higher values of *q* also lead to host populations that recognize a greater proportion of the viral population (see Fig. 6E). Additionally, at higher values of *q* and faster acquisition, more than just the first few spacers are important for immunity (see Fig. 7). This is because the viral strains which the older spacers recognize still exist in the population. See the Supporting Information for analysis of variation in the spacer and protospacer numbers while holding *p* and *q* constant—we do not observe any qualitative differences in dynamics.

## Discussion

We have presented a model of coevolutionary dynamics to analyze the diversification of hosts and viruses. The model demonstrates how an initially small number of host and viral strains can diversify into a dynamic community of many hosts and viruses as anticipated from empirical studies (Heidelberg et al. 2009; Held et al. 2010). In so doing, we confirm theoretically the hypothesis suggested from empirical data that if CRISPR immunity and viral diversification remain in balance, a relatively stable virus and host community may result (Andersson and Banfield 2008; Held et al. 2010). Specifically, the model predicts that diversity over time is maintained by the temporally limited emergence, dominance, and replacement of strains (and coalitions of strains). We observe incomplete sweeps by single strains, the occasional recurrence of rare, older strains that obtain temporary fitness advantages (similar in form to negative frequency-dependent selection), as well as the emergence of coalitions who possess nearly identical phenotypes with distinct spacer genotypes as predicted in Held et al. (2010). The balance of viral and host coevolution occurs despite the fact that the CRISPR system undergoes directed mutation. We find that the hosts generally cannot acquire so many spacers such that the viral population goes extinct. Indeed, viral mutants that can target dominant hosts are under positive selection because their replication will be greater on dominant hosts. Hence, evolutionary changes in viral strain composition drives the change in host strain abundances from abundant to rare and rare to abundant. Moreover, we predict that due to strain replacement, the protospacers matching spacers at the trailing end of host loci are no longer present in dominant members of the viral population, and therefore only the first few spacers contribute significantly to host immunity. The importance of the position of spacers is also correlated to the rate of spacer acquisition. Finally, on the basis of a sensitivity analysis of our model with varying molecular model parameters, we hypothesize that it is the spacer acquisition rate rather than the CRISPR immune failure rate that drives the complexity of the resulting community.

It is important to keep in mind that CRISPR immunity need not function quantitatively, nor necessarily qualitatively, similarly within different host organisms and different ecological contexts. Indeed, the study of CRISPR immunity is in its relative infancy. The possible significance of CRISPRs was first anticipated by bioinformatics studies (Jansen et al. 2002) and a growing suite of bioinformatics tools suggest that up to 40% of all extant bacteria genomes and nearly all archaeal genomes have CRISPR-like regions (Haft et al. 2005; Bland et al. 2007; Edgar 2007; Grissa et al. 2008). What we know about CRISPR function empirically derives largely from the study of *S. thermophilus* (Deveau et al. 2008; Horvath et al. 2008). However, CRISPR-like mechanisms have been reported in a wide variety of hosts, including *E. coli* (Westra et al. 2010), acid mine drainage bacteria (Andersson and Banfield 2008), thermophiles such as *Sulfolobus islandicus* (Held et al. 2010), and microbial mat bacteria (Heidelberg et al. 2009), to name just a few of a growing list of examples. As such, this (or any other) model cannot be considered comprehensive. Nonetheless, developing a multiscale eco-evolutionary CRISPR model sheds light on aspects of host–viral diversification.

The current model has a number of qualitative differences with the limited number of prior efforts to model the CRISPR system. A previous effort to model CRISPR-induced evolutionary dynamics (He and Deem 2010) utilized an immunological-based approach in which viral production is uncoupled from host density (unlike the current model in which viral production is linked explicitly to host density). That model also utilizes a finite allele space, which has advantages in terms of simulation speed, but possible disadvantages in terms of assuming a finite set of possible protospacers. The ecological component presented here is similar to a recent model of CRISPR interactions (Levin 2010) which primarily focused on ecological competition between strains that possess CRISPR immunity versus those that possess envelope resistance to viruses. More generally, we envision future CRISPR models confronting the large number of ecological mechanisms thought to be responsible for coexistence between hosts and viruses, such as competition between hosts with multiple defense mechanisms (Levin 2010), multiple trophic effects (Thingstad and Lignell 1997; Thingstad 2000), modifications to host–virus interaction modes via treatment of implicit and explicit resource modeling (Levin et al. 1977; Weitz and Dushoff 2008; Menge and Weitz 2009) lysogenic life history (Stewart and Levin 1984; Wang and Goldenfeld 2010), and even inclusion of spatial dynamics (Schrag and Mittler 1996; Buckling and Rainey 2002; Heilmann et al. 2010) as one recent CRISPR model has done (Haerter et al. 2011). At the moment, all currently available CRISPR models have features that capture some, but not all, consensus principles of CRISPR immunity. From an empirical perspective, these models are likely to be of greater service when their assumptions are borne out in the particular taxa or ecological conditions of interest.

The current model of coevolutionary dynamics involves a number of assumptions and carries with it a number of caveats. First, we restrict our attention to ecological and molecular parameters that satisfy the following conditions: viruses die out when infecting immune hosts and viruses coexist with nonimmune hosts. Specifically, we choose life-history parameters typically used in models of microbial hosts and their viruses (De Paepe and Taddei 2006). As we showed, when rates of viral mutation are small, it is possible that viral populations may suffer CRISPR-induced extinction. Hence, quantitative parameter values do matter, and efforts to estimate some of the least well-understood parameters may be helpful in testing both the assumptions and predictions of available theories. Next, we have implemented a simplified CRISPR model, in which we ignore the possibility of multiple simultaneous changes in spacer or protospacer states. In doing so, we are making implicit assumptions about the magnitude of directed and undirected mutations that can occur. For example, we do not consider the possibility that viruses reshuffle genomes and/or undergo mutational events distributed on the typical length of a protospacer. We also avoid explicit treatment of other modes of spacer deletion which are certainly more complex than the current treatment of a constant spacer locus size. Both of these topics are important targets for future work. Finally, the degree of resistance of hosts to viruses is thought to depend on the number of spacers, and more importantly, on the degree of similarity between spacer sequences in a host genome and protospacer sequences in the viral/plasmid genome that has been introduced in the cellular cytoplasm (Barrangou et al. 2007). A large body of work in the study of host–pathogen relationships has focused on the genetic determinants of host–parasite outcomes (e.g., (Sasaki 2000; Agrawal and Lively 2002)). The CRISPR system may yet fall into another category, because matches of subsets of alleles impact resistance levels (Barrangou et al. 2007). Understanding how the number of matches between spacers and protospacers and the sequence similarity of matches influences resistance will improve analysis of host–virus dynamics at larger scales.

Adaptive immunity may be a novel finding among bacteria, archaea, and associated viruses: but does the presence of Lamarckian evolution affect population dynamics in ways different than would Darwinian evolution (Bondurianksy and Day 2009)? For microorganisms and their viruses, the time scales of plastic change and evolutionary change can be fast and comparable to ecological time scales. We suggest that a future research goal is to identify if CRISPR mechanisms accelerate the same type of coevolution that one would expect from non-CRISPR mechanisms, or alternatively, generate distinguishing dynamical signatures of coevolution at the level of populations or individual strains. Such a goal should also be accompanied by efforts to evaluate how CRISPR immunity interacts with other types of immune mechanisms (e.g., envelope-based resistance (Levin 2010)). The interest in CRISPR immunity notwithstanding, it is important to keep in mind that CRISPR immunity is one of many defense mechanisms utilized by bacteria and archaea (Hyman and Abedon 2010; Labrie et al. 2010). Hence, efforts to analyze the CRISPR system should also strive to evaluate when and how CRISPR immunity impacts ecosystem structure or function, and evaluate its relative importance compared to other diversification mechanisms.

## Supporting Information

The following supporting information is available for this article:

**Text S1.** Additional analysis of the coevolutionary model and framework.

**Table S1.** Values of viral mutation rate, μ, affect the ability of viral mutants to appear before dying out as well as the length of time required to run simulations.

**Figure S1.** Many host and viral strains exist in the population but most have low abundance.

**Figure S2.** Histogram of host and viral lifetimes.

**Figure S3.** Dynamics of individual host strains change rapidly.

**Figure S4.** New protospacers are rarely incorporated as spacers into hosts.

**Figure S5.** Birth times of host and viral strains.

**Figure S6.** Detailed simulation protocol.

**Figure S7.** Strain mutation rates do not change significantly between sampling time points.

**Figure S8.** The effect of spacer and protospacer numbers on the dynamics.

**Figure S9.** The effect of spacer and protospacer numbers on relative immunity.
