# Supplementary material for: MULTISCALE MODEL OF CRISPR-INDUCED COEVOLUTIONARY DYNAMICS: DIVERSIFICATION AT THE INTERFACE OF LAMARCK AND DARWIN
Source: Evolution. 2012 Jul;66(7):2015–29. doi: 10.1111/j.1558-5646.2012.01595.x (PMC3437473; doi:10.1111/j.1558-5646.2012.01595.x)
Supplement: Supplementary file 1 [file evo0066-2015-SD1.pdf]

Supplementary Information

Multi-scale Model of CRISPR-induced Coevolutionary Dynamics: Diversification at the Interface of Lamarck and Darwin

Lauren M. Childs[1], Nicole L. Held[2], Mark J. Young[3], Rachel J. Whitaker[4,*], Joshua S. Weitz[5,*]

1 School of Biology and School of Mathematics, Georgia Institute of Technology, 310 Ferst Dr, Atlanta, GA 30332, United States of America, lauren.childs@biology.gatech.edu
2 Department of Microbiology, University of Illinois at Urbana-Champaign, 601 S. Goodwin Avenue, Urbana, IL 61801, United States of America, nheld2@illinois.edu
3 Thermal Biology Institute and Department of Plant Sciences and Plant Pathology, Montana State University, 307 Plant Bioscience Building, Bozeman, MT 59717, United States of America, myoung@montana.edu
4 Department of Microbiology and Institute for Genomic Biology, University of Illinois at Urbana-Champaign, 601 S. Goodwin Avenue, Urbana, IL 61801, United States of America, rwhitaker@life.illinois.edu
5 School of Biology and School of Physics, Georgia Institute of Technology, 310 Ferst Dr, Atlanta, GA 30332, United States of America, jsweitz@gatech.edu
*** Corresponding authors**

Host-virus coexistence given a wide range of ecological and molecular parameters

We first analyze simplified versions of the dynamics in order to understand if and under what conditions hosts and viruses can coexist in the absence of *de novo* generation of novel genome states. To do so, consider an environment containing a single host genotype and a single viral genotype whose population densities are denoted as N and V and whose genome states are denoted as S and G. First, assume that $M(S, G) = 0$, in other words, that the host is not immune to the virus via CRISPR mechanisms. In that case, the ecological dynamics of this system can be written as (see Equations (1-2) in the main text)

$$\frac{dN}{dt} = rN\left(1 - \frac{N}{K}\right) - (1-q)\phi NV \quad \text{(S1)}$$

$$\frac{dV}{dt} = \beta(1-q)\phi NV - \phi NV - mV \quad \text{(S2)}$$

The steady state population densities can be found by setting $\mathrm{d}N/\mathrm{d}t = 0$ and $\mathrm{d}V/\mathrm{d}t = 0$. We find three possible steady states: (i) a trivial equilibrium where both hosts and viruses are absent ($N^* = 0, V^* = 0$); (ii) an equilibrium where hosts are at carrying capacity and viruses are extinct ($N^* = K, V^* = 0$); (iii) a coexistence equilibrium where both hosts and viruses are present. Coexistence only occurs for certain regimes of parameters. We find that $N^* = \frac{m}{\phi(\beta-1-\beta q)}$ and that $V^* = \frac{r(1-N^*/K)}{(1-q)\phi}$. The two conditions for coexistence are: (i) $\beta(1-q) > 1$; (ii) $\frac{K\phi(\beta(1-q)-1)}{m} > 1$. However, the typical number of virions produced is on the order of dozens, hundreds or even thousands. Further, rates of spacer acquisition are presumed to be quite small, i.e., $q \ll 1$. Hence, in practice, the first condition is nearly always met. This means that the virions produced by successful infections are far greater than those viruses lost due to unsuccessful infections (when a host is not immune). The second condition is typical of host-virus models. For example, if we approximate $\beta(1-q)-1 \approx \beta$ then the second term is the basic reproductive number, $R_0 \equiv K\phi\beta/m$ for a virus infecting a host population at its carrying capacity, $N^* = K$ given an adsorption constant ϕ, burst size β and extra cellular mortality rate m. These results suggest that in order to model realistic dynamics we should focus on parameter values for which $R_0 > 1$.

Next, consider an environment with a single host genotype and a single viral genotype and assume that $M(S, G) = 1$. In other words, the host is immune to the virus via CRISPR mechanisms. In that case, the ecological dynamics of this system can be written as (see Equations (1-2) in the main text)

$$\frac{\mathrm{d}N}{dt} = rN\left(1 - \frac{N}{K}\right) - p\phi NV \tag{S3}$$

$$\frac{\mathrm{d}V}{dt} = \beta p\phi NV - \phi NV - mV \tag{S4}$$

As before, the steady state population densities can be found by setting $\mathrm{d}N/\mathrm{d}t = 0$ and $\mathrm{d}V/\mathrm{d}t = 0$. Ignoring the trivial and host-only equilibria, we find by analogy with the prior results that the coexistence equilibria occurs when $N^* = \frac{m}{\phi(p\beta-1)}$ and $V^* = \frac{r(1-N^*/K)}{p\phi}$. Hence, the two conditions for coexistence are: (i) $p\beta > 1$; (ii) $\frac{K\phi(p\beta-1)}{m} > 1$. However, the failure rate of the CRISPR system is thought to be quite low, for example, efficiency of plating experiments suggest that p (the success rate of viruses infecting immune hosts) is on the order of 10^{-5}. Hence $p\beta$ will be much less than 1, and so more viruses die by infecting hosts than they do by exploiting hosts. In such a regime, coexistence is impossible, suggesting that a virus population cannot perpetually coexist on a host immune to it in the absence of other mechanisms that facilitate coexistence.

In summary, we restrict our attention to ecological and molecular parameters which satisfy the following two conditions: (i) viruses die out when infecting immune hosts; (ii) viruses coexist with non-immune hosts. Given small error rates and large burst sizes, these conditions can be written compactly as: $\frac{K\phi\beta}{m} > 1$ and $\frac{1}{1-q} < \beta < \frac{1}{p}$.

Mutant hosts and viruses arising from directed and undirected mutation, respectively, can successfully invade a resident population

The basis for the *de novo* generation of novelty in this model is the dynamic updating of spacer, S, and protospacer, G, states. Here, we show analytically that novel genotypes of both hosts and viruses can have positive per-capita growth rates when rare. Consider first the host-only equilibrium, in which $N^* = K$ and $V^* = 0$. The question we ask is whether a new virus population will increase in numbers if the resident host population does not possess immunity to it. The new viral population will experience a per-capita growth rate of

$$\beta(1-q)\phi K - \phi K - m, \tag{S5}$$

based on Equation (S2). Note that this growth rate is positive given the biological conditions of interest described above. Hence, coexistence of viruses with hosts that do not possess CRISPR immunity implies the invasion of a rare virus population in a host-only environment.

Next, we ask whether a mutant host population can invade a system comprised of resident hosts and viruses. The mutant host has immunity to the viruses whereas the resident host does not. The mutant host population will obey:

$$\frac{\mathrm{d}N'}{dt} = rN'\left(1 - \frac{N+N'}{K}\right) - p\phi N'V. \tag{S6}$$

We can evaluate the success of the invasion by recalling that $N^* = m/\left(\phi\left(\beta - 1 - \beta q\right)\right)$ and that $V^* = \frac{r(1-N^*/K)}{(1-q)\phi}$. Hence, the per-capita growth rate of the mutant host given resident hosts with population N^* and resident viruses with population V^* is:

$$r\left(1 - \frac{N^*}{K}\right) - \frac{rp\left(1 - \frac{N^*}{K}\right)}{(1-q)} \tag{S7}$$

The per-capita growth rate of the mutant host will be positive so long as $p/(1-q) < 1$. For $p \ll 1$ and $q \ll 1$, this is certainly satisfied, and so mutant hosts will invade as is expected. Because of the principle of competitive exclusion, the resident host will die out, and so the new steady state will be one of $N^* = 0, N'^* = K, V^* = 0$.

In summary, if we consider a mutation-limited system in which ecological dynamics are much faster than evolutionary dynamics then we expect: (i) a virus to stably coexist with a host which possesses no immunity to it; (ii) a mutant host to emerge, leading to the death of the resident host and the resident virus; (iii) a new virus which is added to the system for which there is no standing immunity will invade leading back to (i) above. Together, these results suggest a mechanism by which novelty will be introduced and a mechanism by which it will be eliminated. In reality, ecological and evolutionary time scales are not always so separable. It is possible that more complex patterns of diversification, including increases of diversity toward a dynamic steady state, may occur.

Detailed Simulation Protocol

The initial state of our simulations consist of a single susceptible host and a single virus. We begin our simulation procedure by estimating the mutation rate of each strain (see Figure S6). The mutation rate, μ_{N_i}, of each host strain depends on its abundance (N_i), the abundance of all viral strains ($\sum_j V_j$), the interaction rate (ϕ_{ij}), and the spacer acquisition rate (q), via $\mu_{N_i} = \sum_j \phi_{ij} N_i V_j q$. The mutation rate, μ_{V_j}, of each viral strain depends on its abundance (V_j), the burst size (β), the per-protospacer viral mutation rate (μ), the interaction rates (ϕ_{ij}), and the probability of viral lysis (p when the host is immune and $1-q$ when the host is susceptible), via $\mu_{V_j} = \sum_i \beta\mu\phi_{ij} N_i V_j (1-q)$ when the host is susceptible or $\mu_{V_j} = \sum_i \beta\mu\phi_{ij} N_i V_j p$ when the host is immune.

We recalculate the mutation rate for each strain after each event (strain extinction, strain mutation, or data recording) and assume it is constant until the next event. Although the values of N_i and V_j are continuously varying in time, we treat the continuous time-varying mutation rates as piecewise smooth mutation rates. As our time intervals between events are very short the mutation rates change very little over each period. We demonstrate this by comparing the mutation rate of each strain before and after every event: $\frac{\mu(t+\delta t)}{\mu(t)}$ (See Figure S7). The majority of the comparisons ($> 90\%$) are within 0.05 of 1 and all fall between 0.5 and 2.5. This distribution is narrow and centered around 1 indicating that the mutation rates do not change significantly between events, i.e., they are effectively constant.

We use the Gillespie algorithm to determine the time until the next mutation event, t_m. We compare this time with the interval for data recording, t_{record} (1 hr for all simulations in this paper), to determine which event occurs next and proceed as follows (See Figure S6).

If the time interval, t_m, is greater than the time interval until the next data recording point, t_{record}, then the next predicted event is a data recording event. The system of ODEs is deterministically solved in Matlab using ode45 for the time interval $[t_{\text{current}}, t_{\text{current}} + t_{\text{record}}]$. If the system of ODEs stops via an event function with $t < t_{\text{current}} + t_{\text{record}}$, there is a strain extinction event prior to the predicted recording event. Our event function stops the ODE solver whenever the population density of any strain falls below our critical population threshold, ρ_c. A strain extinction event results in the removal of the ODE associated with the strain whose density fell below the threshold. If the system of ODEs stops with $t = t_{\text{current}} + t_{\text{record}}$, data is recorded and the ODE system does not change in size. Regardless, the mutation rates are recalculated.

If the time interval, t_m, is less than the time interval until the next data recording point, t_{record}, then the next predicted event is a mutational event. The system of ODEs is deterministically solved in Matlab using ode45 for the time interval $[t_{\text{current}}, t_{\text{current}} + t_m]$. If the system of ODEs stops via an event function with $t < t_{\text{current}} + t_m$, there is a strain

extinction event prior to the predicted mutational event that results in the removal of the ODE associated with the strain whose density fell below the threshold, as explained above. If the system of ODEs stops with $t = t_{\text{current}} + t_m$, the predicted mutational event will occur. As a mutational event will produce a new strain, an ODE is added to the system.

Mutational events occur as follows. In the case of host acquisition of a spacer, a given host strain is probabilistically selected to acquire a new spacer in proportion to its instantaneous rate of successful defense events. The viral strain from which it will draw a protospacer is chosen probabilistically in proportion to its interaction rates taking into account immunity. Once the viral strain from which the host will draw a spacer is selected, then the protospacer is chosen randomly from the set of all protospacers in that virus. Provided no other host strain has an identical set of spacers, the new host strain is included in the simulation with a population density 10% greater than the extinction threshold, ρ_c. The parent host strain remains in the simulation without a change in its population density.

Similarly, in the case of viral mutation, a given strain of virus is probabilistically selected to undergo a mutation event in proportion to the instantaneous growth rate of that strain. The protospacer to mutate is chosen randomly from the set of all protospacers in the chosen viral strain. The newly added protospacer is always novel so the new viral strain is included in the simulation with a population density 10% greater than the extinction threshold. The parent viral strain remains in the simulation without a change in its population density.

Once one of the following events has taken place – (i) a host or virus strain goes extinct; (ii) a mutation event occurs; or (iii) the simulation reaches a defined time point for data output – the mutation rate of each strain is recalculated, using the formulas which depend on abundances given above. This entire process is repeated until one of the following occurs: all host strains go extinct, all viral strains go extinct, or the simulation reaches the maximum running time (2,500 hours for the results presented in this paper).

Viral population size as a function of q

When hosts are unable to acquire spacers ($q = 0$), the host population density and viral population density settle to the top-down controlled steady state described previously such that host density is at a minimum ($N^* = \frac{m}{(\beta-1)\phi}$) and viral density is exactly as predicted ($V^* = \frac{r}{\phi}\left(1 - \frac{N^*}{K}\right)$). When $q = 1$, hosts gain immunity at every interaction so the viral population goes extinct, leading to a maximum in host density at the carrying capacity. Thus as the spacer acquisition parameter q is increased from 0 and hosts more rapidly acquire spacers, it may be expected that the total viral population density will decrease. However, we observe that the viral population size increases as the number of host increases, a result of increasing q when $q \ll 1$. From our exploration of changing p and q with $p \ll 1$ and $q \ll 1$, it appears the viral population size is a unimodal function

of q or at least the dynamics are complicated, depend on host diversity and do not simply decrease monotonically.

Now, let us imagine that q is small, such that multiple host and viral strains coexist. In general such infection patterns are complex. Let us consider the simple case such that there are two host strains with two viral strains that can only infect each host type exclusively (i.e. V_1 can only infect N_1 and V_2 can only infect N_2). In such a case, we have the following system of equations:

$$
\begin{aligned}
dN_1/dt &= rN_1(1-\frac{N_1+N_2}{K})-(1-q)\phi N_1V_1-p\phi N_1V_2 \\
dN_2/dt &= rN_2(1-\frac{N_1+N_2}{K})-(1-q)\phi N_2V_2-p\phi N_2V_1 \\
dV_1/dt &= \beta(1-q)\phi N_1V_1+\beta p\phi N_2V_1-\phi(N_1+N_2)V_1-mV_1 \\
dV_2/dt &= \beta(1-q)\phi N_2V_2+\beta p\phi N_1V_2-\phi(N_1+N_2)V_2-mV_2
\end{aligned}
$$

Solving this system gives similar results: $N_1^*=\frac{m}{((1+p-q)\beta-2)\phi}$, $N_2^*=\frac{m}{((1+p-q)\beta-2)\phi}$, $V_1^*=\frac{r}{\phi(1+p-q)}\left(1-\frac{N_1^*+N_2^*}{K}\right)$, and $V_2^*=\frac{r}{\phi(1+p-q)}\left(1-\frac{N_1^*+N_2^*}{K}\right)$. So that the total viral density in this particular scenario with two hosts strains is $V_1^*+V_2^*=V_{tot}^*=\frac{2r}{\phi(1+p-q)}\left(1-\frac{N_1^*+N_2^*}{K}\right)$.

This leads to the condition that, at least in this specific example, the viral density with two host strains ($s=2$) is greater than with one host strain ($s=1$): $V_{tot}^*(s=2)>V_{tot}^*(s=1)$ if $\frac{2r}{\phi(1+p-q)}\left(1-\frac{2m}{((1+p-q)\beta-2)\phi K}\right)>\frac{r}{\phi(1-q)}\left(1-\frac{m}{((1-q)\beta-1)\phi K}\right)$.

Although these formulas seem complicated, we can simplify them significantly by observing that $p,q\ll 1$ and $\beta\gg 1$. Using these simplifications our steady states become: $N_1^*=\frac{m}{\beta\phi}$, $N_2^*=\frac{m}{\beta\phi}$, $V_1^*=\frac{r}{\phi}\left(1-\frac{N_1^*+N_2^*}{K}\right)$, and $V_2^*=\frac{r}{\phi}\left(1-\frac{N_1^*+N_2^*}{K}\right)$. Additionally, the condition for an increase in viral density, $V_{tot}^*(s=2)>V_{tot}^*(s=1)$, becomes $1>\frac{3m}{K\phi\beta}$.

Now consider there are s host strains with s viral strains that can only infect each host type exclusively (i.e. V_1 can only infect N_1, V_2 can only infect N_2, V_3 can only infect N_3 and so forth). Going through the same calculation, we find that $V_{tot}^*(s)>V_{tot}^*(s=1)$ when $\frac{sr}{\phi(1+(s-1)p-q)}\left(1-\frac{sm}{((1+(s-1)p-q)\beta-s)\phi K}\right)>\frac{r}{\phi(1-q)}\left(1-\frac{m}{((1-q)\beta-1)\phi K}\right)$. Again consider the scenario where $p,q\ll 1$, then $V_{tot}^*(s)>V_{tot}^*(s=1)$ if $s\left(1-\frac{sm}{(\beta-s)\phi K}\right)>1-\frac{m}{(\beta-1)\phi K}$. Using the parameters from our model ($\beta=50$, $\phi=10^{-7}$, $K=10^{5.5}$, $m=0.1$), this holds from $s=2$ until $s\sim 11$.

Hence, adding diversity of hosts, can allow more viral types to infect distinct host types, leading to an increase in viral population density. In the specific scenario considered here V_{tot} is a unimodal function of q. Although the dynamics of our eco-evolutionary models are complicated, we anticipate that there will exist a non-trivial relationship between q and viral density, likely unimodal.

Dependence of coevolutionary dynamics on the number of spacers and protospacers

Here, we consider the effect of varying the number of spacers per host and the number of protospacers per virus on the outcomes of the multi-scale coevolutionary model. In varying these parameters, we considered values ranging from 4 spacers per host to 8 spacers per host and from 6 protospacers per virus to 10 protospacers per virus. We do not increase the number of protospacers per virus beyond 10 due to computational constraints. In keeping with the experimental observation that there are more protospacers per virus, the number of protospacers is always greater than the number of spacers per host by at least two. We find that changing the number of spacers or protospacers does not affect the dynamics significantly. As the number of spacers per host increases, the hosts have immunity to a greater fraction of the viral population. Since there are more spacers per host, there are more opportunities for each host strain to have immunity to viruses (see Figure S8e). As the number of spacer per host and protospacer per virus increases, the number of viral strains decreases (see Figure S8d). At the same time, the population size of both hosts and viruses increases (See Figure S8a-b). In contrast to increasing q, the increased viral density seen here arises from increased host population density rather than an increase in the number of host strains. Similarly, we find that the initial spacers dominate the contributions to relative immunity regardless of the number of spacers and protospacers (see Figure S9).

Viral mutation rate, μ	Percent of replicates evading initial die-out (in first 200 hours)	Relative running time (to $\mu = 10^{-8}$)
10^{-6}	100	229.4
$5 * 10^{-7}$	100	109.1
10^{-7}	90	12.2
$5 * 10^{-8}$	74	3.6
10^{-8}	25	1

Table S1. Values of viral mutation rate, μ, affect the ability of viral mutants to appear before dying out as well as the length of time required to run simulations.

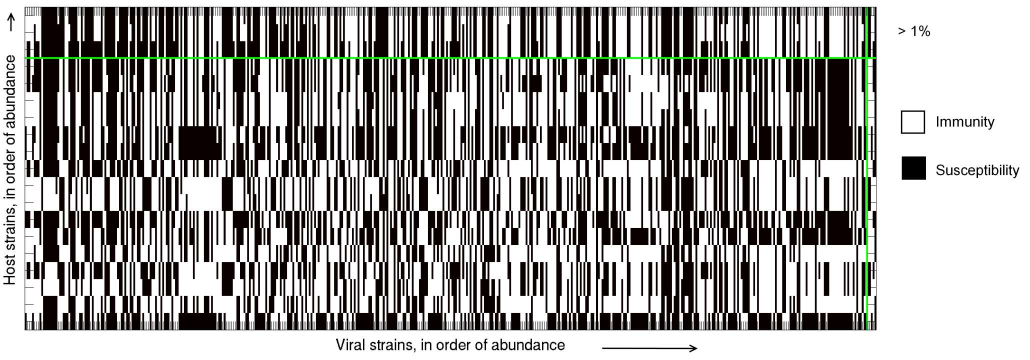

Figure S1. Many host and viral strains exist in the population but most have low abundance.
Dominant host strains are immune (white squares) to dominant viral strains at $t = 1500$ while many low abundance host strains remain susceptible (black) to viral strains. Strains are listed in order of abundance with host strains on the vertical axis and viral strains on the horizontal axis. More abundant host strains are at the top, and more abundant viral strains are to the right. The green lines distinguish between strains composing more than 1% of the population (host - above, viral - right) and strains composing less than 1% of the population (host - below, viral - left). The color of the box indicates whether there is immunity (white) or susceptibility (black). The immunity matrix shown is from a single representative simulation.

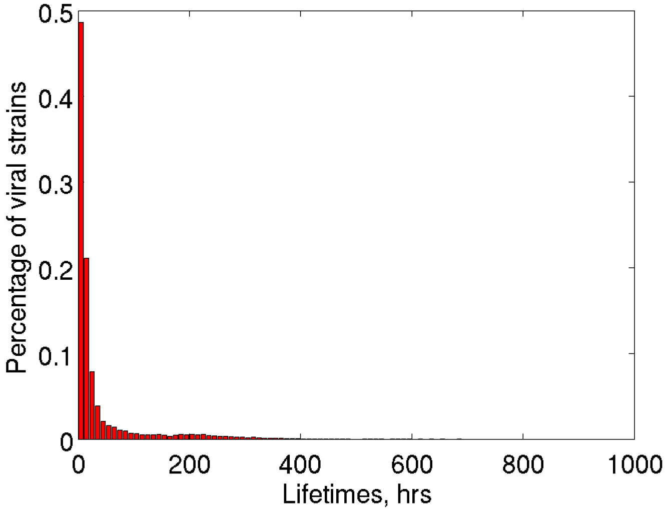

(a) Lifetimes of Viruses

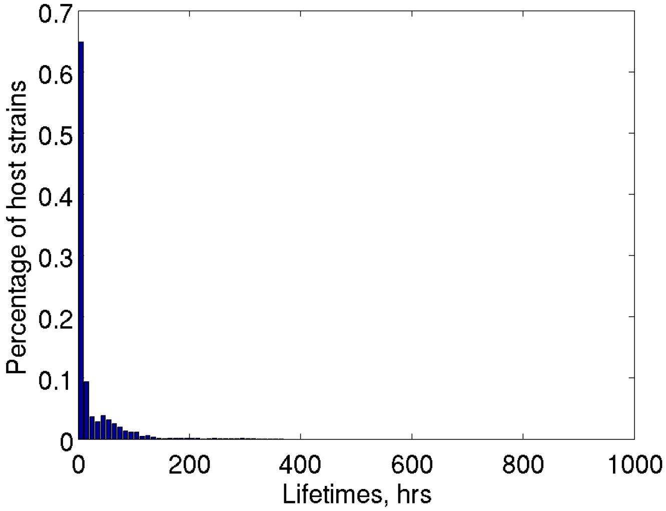

(b) Lifetime of Hosts

Figure S2. Short lifetimes of hosts and viruses are consistent with the maintenance of strain diversity through continual replacement with new types.
Strain lifetimes of (A) viruses and (B) hosts are predominantly short although a few strains have lifetimes of up to 1000 hrs (computed from 100 replicate simulations).

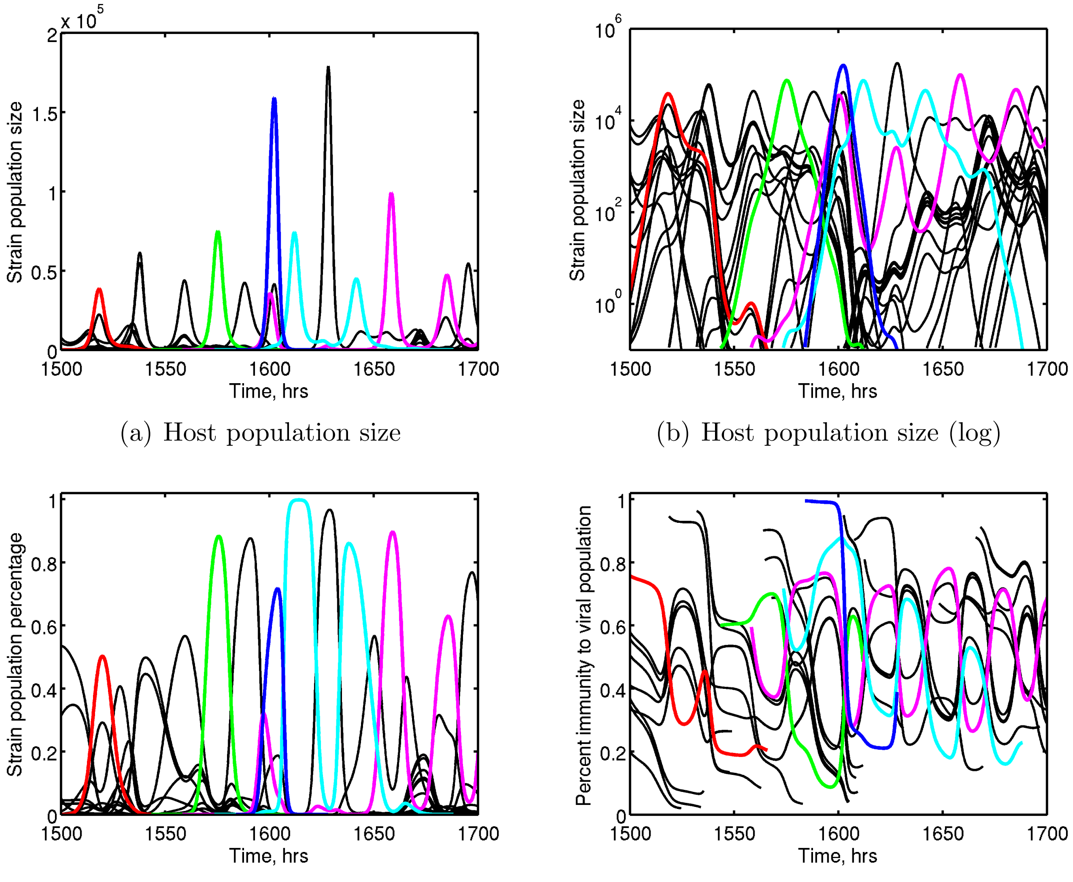

(a) Host population size (b) Host population size (log)

(c) Percentage of host population (d) Percent immunity to virus population

Figure S3. Dynamics of individual host strains changes rapidly. Fluctuations in the number of each host strain (A),(B) and the percentage of the total host population (C) arise from the amount of immunity to the viral population each host strain possess (D). Each line represents a different strain, and the corresponding strains in each plot have the same color. Note: All host strains comprising greater than 1% of the host population are included. When lines begin or end abruptly, the strain has emerged from or fallen below the 1% threshold. The dynamics are from a single representative simulation.

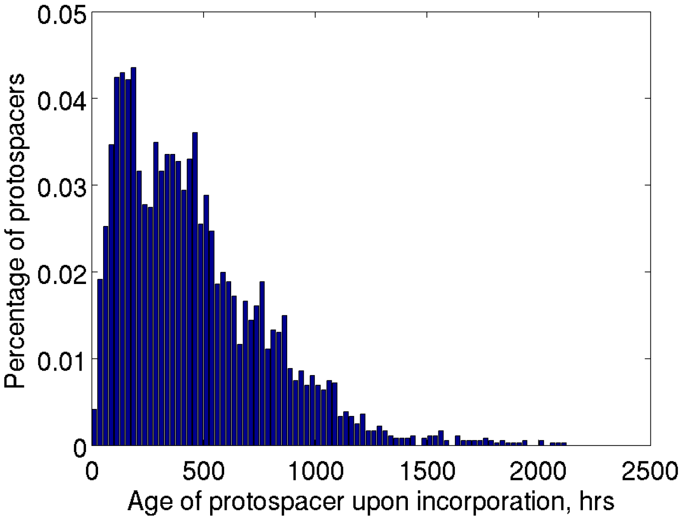

Figure S4. New protospacers are rarely incorporated as spacers into hosts. At the time of their incorporation into a host, most protospacers (average over 100 replicate simulations) have been in the simulation between 100 and 2000 hours. As the average time between virus births is less than one hour, this indicates that most protospacers which are incorporated are not new (see Figure S5).

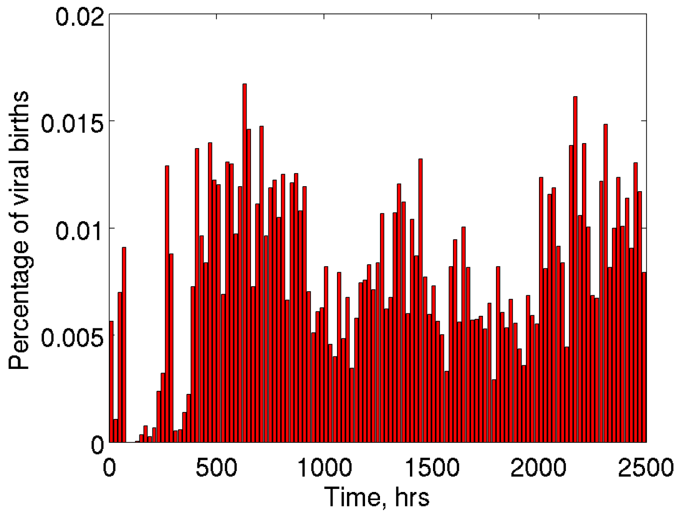

(a) Birth times of Viruses

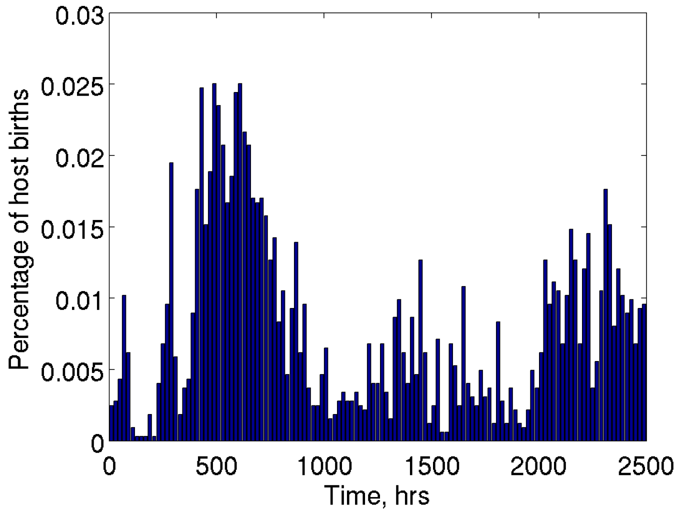

(b) Birth times of Hosts

Figure S5. Birth times of host and viral strains
Histogram of the number of births of viral (A) and host (B) strains during the course of the simulation. Increased viral and host births are correlated.

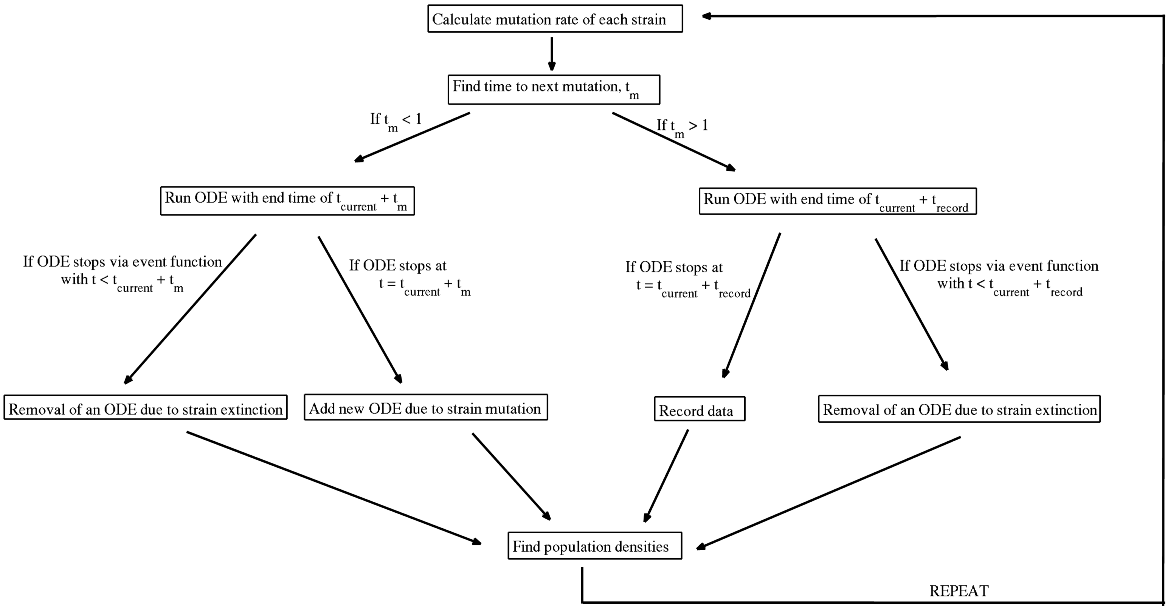

Figure S6. Simulation Method
The simulation proceeds until one of the following events: (i) a host or virus strain goes extinct; (ii) a mutation event occurs; or (iii) the simulation reaches a defined time point for data output. Once this occurs the population densities are used to recalculate the mutation rate of each strain.

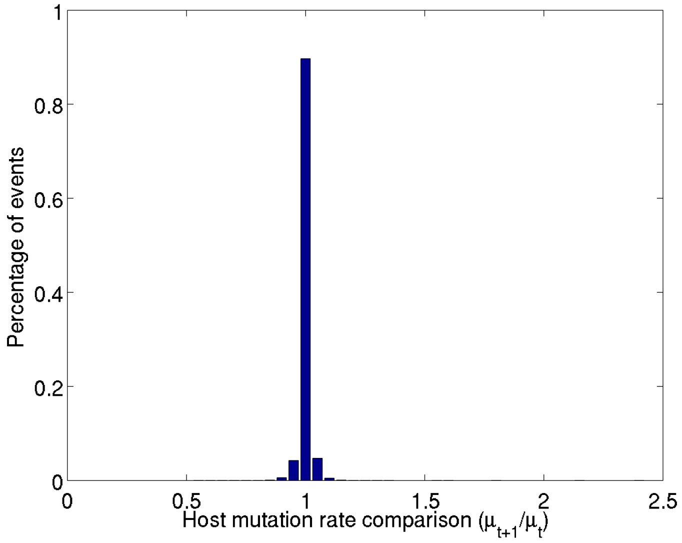

(a) Host

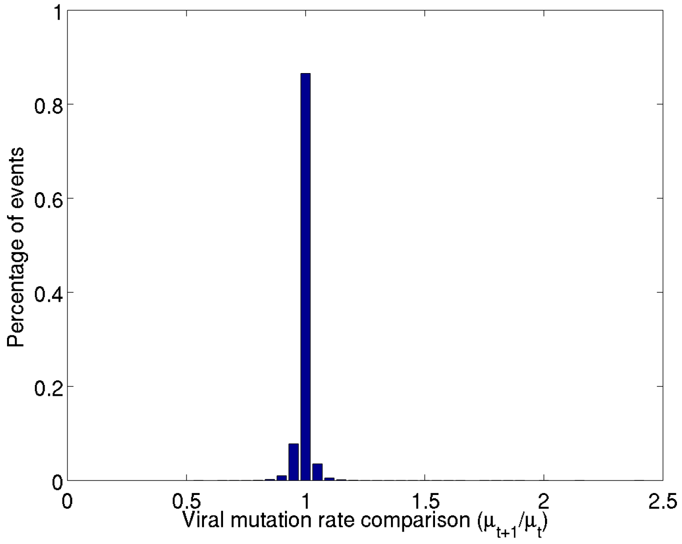

(b) Viral

Figure S7. Strain mutation rates do not change significantly between sampling time points.
The mutation rate of strains, (A) host and (B) viral, is recalculated after each event. The relative difference between the mutation rate at one event point t and the next event point $t + \delta t$ is plotted. The mutation rate has not changed significantly over these intervals and thus the distribution is centered around 1. The mutation rates are computed from a single representative simulation (out of 100 replicates).

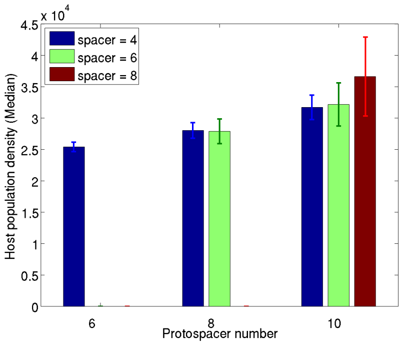

(a) Host Population Density

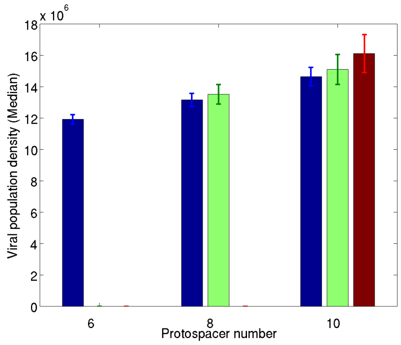

(b) Viral Population Density

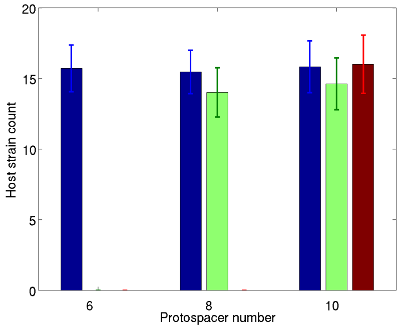

(c) Host Strain Count

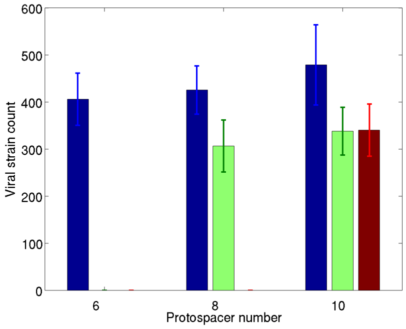

(d) Viral Strain Count

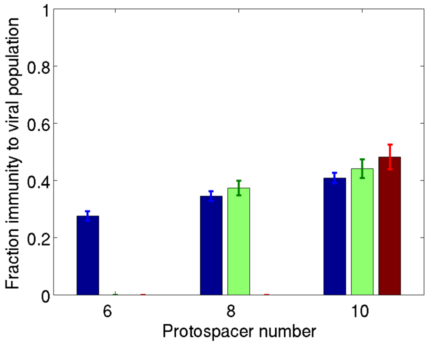

(e) Immunity to Viral Population

Figure S8. Spacer and protospacer numbers have little effect on the dynamics. The number of spacers per host is varied from 4 to 8 and the number of protospacers per virus is varied from 6 to 10. Values of protospacers per virus are grouped on the x-axis. Values of spacers per host have identically colored bars (Blue represents spacer = 4; green represents spacer = 6; red represents spacer = 8.) All bars represent median of 100 replicates and the lines represent standard error.

As the number of protospacers per virus and spacers per host increases, host population density (A) increases, viral population density (B) increases, host strain counts (C) remain unchanged, viral strain counts (D) decrease, and the fraction of the viral population the hosts are immune to (E) increases.

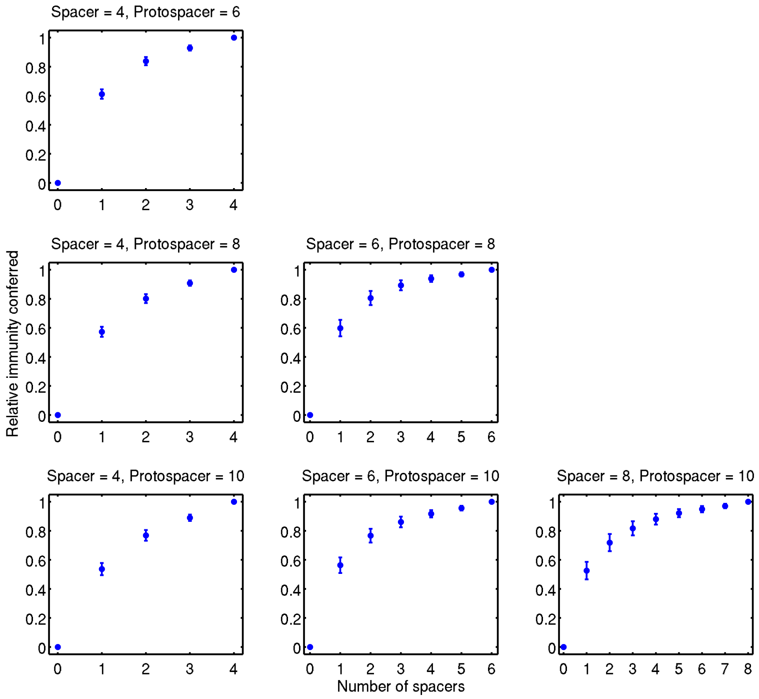

Figure S9. Spacer and protospacer numbers have little effect on relative immunity. Relative immunity conferred by the newest n spacers in the locus compared to the immunity from the full locus of spacers. Mean (circles) and standard deviation (error bars) were computed for 100 replicates averaged over the time points after the locus is filled with spacers. Immunity is determined by calculating what percentage of the viruses the most recent n spacers from all hosts can match, where $n = 1, 2, ...$ Relative immunity is the percentage of viruses the most recent n spacers from all hosts can match compared to the percentage of viruses the full spacer locus matches. The number of spacers per host is varied from 4 to 8 and the number of protospacers per virus is varied from 6 to 10. All bars represent 100 replicates and the lines represent standard error.
